# Facilitation of Co-Metabolic Transformation and Degradation of Monochlorophenols by *Pseudomonas* sp. CF600 and Changes in Its Fatty Acid Composition

**DOI:** 10.1007/s11270-016-2775-5

**Published:** 2016-02-13

**Authors:** Agnieszka Nowak, Agnieszka Mrozik

**Affiliations:** Department of Biochemistry, Faculty of Biology and Environmental Protection, University of Silesia, Jagiellońska 28, 40-032 Katowice, Poland

**Keywords:** *Pseudomonas* sp. CF600, Monochlorophenols, Co-metabolism, Dioxygenases, Fatty acids

## Abstract

In this study, co-metabolic degradation of monochlorophenols (2-CP, 3-CP, and 4-CP) by the *Pseudomonas* sp. CF600 strain in the presence of phenol, sodium benzoate, and 4-hydroxybenzoic acid as an additional carbon source as well as the survival of bacteria were investigated. Moreover, the changes in cellular fatty acid profiles of bacteria depending on co-metabolic conditions were analyzed. It was found that bacteria were capable of degrading 4-CP completely in the presence of phenol, and in the presence of all substrates, they degraded 2-CP and 3-CP partially. The highest 2-CP and 3-CP removal was observed in the presence of sodium benzoate. Bacteria exhibited three various dioxygenases depending on the type of growth substrate. It was also demonstrated that bacteria exposed to aromatic growth substrates earlier degraded monochlorophenols more effectively than unexposed cells. The analysis of fatty acid profiles of bacteria indicated the essential changes in their composition, involving alterations in fatty acid saturation, hydroxylation, and cyclopropane ring formation. The most significant change in bacteria exposed to sodium benzoate and degrading monochlophenols was the appearance of branched fatty acids. The knowledge from this study indicates that *Pseudomonas* sp. CF600 could be a suitable candidate for the bioaugmentation of environments contaminated with phenolic compounds.

## Introduction

Developments in industry and agriculture would not be possible without chemical compounds, like solvents, wood preservatives, pesticides, dyes, or disinfectants. For the production of many of these chemicals, different chlorophenols are commonly used. The global production of chlorophenols is 90,000 t/year, including 60,000 t of lower chlorinated phenols (Veenagayathri and Vasudevan 2013). Furthermore, large amounts of these compounds, especially monochlorophenols, are released into the environment as by-products of the pulp-bleaching process in the paper industry and the chlorination of wastewater and drinking water (Roy et al. 2004; Ge et al. 2006). Due to their acute toxicity, chlorophenols are considered to be priority pollutants by the World Health Organization (WHO), Unites States Environmental Protection Agency (USEPA), and the European Union (EU).

Among all chlorophenols, monochlorophenols are the most mobile in the environment because of their relatively high solubility in water (Czaplicka 2004). They can easily migrate within soil and aqueous environments and therefore contaminate soil, water, groundwater, and sediments. This is the reason why effective methods for their detoxification and/or degradation have been developed. One of them is microbiological degradation with the following three major steps: hydroxylation of monochlorophenols to chlorocatechols, aromatic ring cleavage, and further oxidation of aliphatic compounds to intermediates of central metabolic pathways. The key reaction in this process is aromatic ring cleavage catalyzed by intradiol or extradiol dioxygenases (EC 1.13.11) (Haddock 2010). Intradiol cleavage via the *ortho-*pathway is catalyzed by catechol 1,2-dioxygenase (EC 1.13.11.1). The cleavage reaction product is 3-chloromuconate transformed to *cis*-dienolactone and further to maleylacetate. *Meta*-cleavage of chlorocatechols catalyzed by catechol 2,3-dioxygenase (EC 1.13.11.2) generally results in incomplete degradation to dead end metabolite 5-chloro-2-hydroxymuconic semialdehyde (Arora and Bae 2014). To enhance the degradation of chlorophenols by bacteria, additional carbon sources are commonly applied. Co-metabolism refers to the degradation of two or more compounds, where the biotransformation of the barely degradable compound (co-metabolite) depends on the presence of an additional co-substrate serving as a carbon and energy source. This phenomenon reflects the processes which take place in a natural environment, where a single contaminant is rather rare. Traditionally, the growth substrates have been divided into the following two major groups: conventional carbon sources such as glucose (Bhatkal et al. 2012), sodium glutamate (Wang and Loh 2001), or yeast extract (Fakhruddin and Hossain 2007) and compounds structurally similar to the non-growth substrate (Baggi et al. 2002; Lee and Lee 2007). The conventional carbon sources support cell growth and increase biomass concentration, while structurally analogous compounds induce the enzymes of the co-metabolite metabolic pathways. The development of bioremediation methods based on co-metabolism could allow the counteraction of the accumulation of barely degradable compounds in the environment.

In response to exposure to harmful substances, bacteria can change their membrane lipid composition and integrity. Adaptive mechanisms such as changes in the length of fatty acids, *cis/trans* isomerization, changes in the degree of saturation of fatty acids, the increase in branched and cyclopropane fatty acid content, or the modification of the lipid to protein ratio have been well documented in many species of bacteria (Heipieper et al. 2003; Mrozik et al. 2005; Segura et al. 2010).

The aim of this study was to investigate the co-metabolic degradation of monochlorophenols by the *Pseudomonas* sp. CF600 strain in the presence of phenol, sodium benzoate, and 4-hydroxybenzoic acid as an additional carbon source in batch liquid cultures. Moreover, changes in the cellular fatty acid methyl ester (FAME) profiles of bacteria depending on co-metabolic conditions were analyzed.

## Materials and Methods

### Bacterial Strain and Culture Conditions

The bacterial strain used in this study was *Pseudomonas* sp. CF600, kindly provided by Prof. V. Shingler from the Department of Cell and Molecular Biology, Umeå University, Sweden. It is deposited in the Culture Collection of the University of Goteborg (no. 32333).

Bacteria were grown in a mineral salt medium (Mrozik et al. 2007) containing phenol (P) at a concentration of 282 mg l^−1^, sodium benzoate (SB) 432 mg l^−1^, or 4-hydroksybenzoic acid (4-HB) 414 mg l^−1^ in 500-ml flasks on a rotary shaker (130 rpm) at 30 °C. The initial number of bacterial cells inoculated to the medium was 5 · 10^8^ ml^−1^. In order to examine the rate of degradation of P, SB, or 4-HB and the induction of enzymes involved in the mineralization of these compounds, cells were adapted to each substrate by transferring them three successive times on the same substrate, using each time an initial cell number adjusted to 5 · 10^8^ cells ml^−1^. For the co-metabolic degradation study, bacteria were grown in a mineral salt medium in the presence of SB (432 mg l^−1^), 4-HB (414 mg l^−1^), or P (282 mg l^−1^) as a growth substrate and 2-chlorophenol (2-CP), 3-chlorophenol (3-CP), or 4-chlorophenol (4-CP) at a concentration of 130 mg l^−1^ as a co-metabolite. It was the highest concentration of chlorophenols which bacteria were able to degrade. To determine which growth substrate accelerates co-metabolic degradation of monochlorophenols, bacteria were incubated in the mineral salt medium with appropriate substrate and monochlorophenol for 24 h. To estimate the influence of long-term exposure of *Pseudomonas* sp. CF600 on its ability to co-metabolically degrade monochlorophenols, cells were cultured in the medium containing a mixture of each monochlorophenol and selected growth substrate for 7 days. Results of our previous experiments on co-metabolic degradation of chlorophenols in dependence on the different molar ratio of the growth to the non-growth substrate (1:1, 2:1, 3:1, 4:1, and 5:1) indicated that the optimal ratio was 3:1.

The microbial counts were determined by the dilution plate count technique using nutrient agar. The inoculated plates were incubated at 30 °C for 48 h. The number of bacteria was expressed as log CFU ml^−1^. Data are representative of three individual experiments.

### Determination of Aromatic Compound Concentration

The aromatic compounds were determined by a Merck Hitachi HPLC equipped with an Ascentis^®^ Express C18 HPLC Column (100 × 4.6 mm), an Opti-Solw^®^ EXP precolumn, and a DAD detector (Merck Hitachi). The mobile phase was the mixture of acetonitrile, methanol, and 1 % acetic acid (20:20:60, *v*/*v*). The flow rate was 1 ml min^−1^. Chemical compounds in the supernatant were identified and quantified by comparing HPLC retention times and UV-visible spectra with external standards. The detection wavelength was set at 272 nm for the detection of P, SB, 2-CP, 3-CP, and 4-CP and at 260 nm for 4-HB.

### Enzymes Activity Assays

For the preparation of crude extract for dioxygenase activity, assay cells were harvested in the late exponential phase of growth by centrifugation at 4612*g* for 20 min at 4 °C. The cells were washed with a 50-mM phosphate buffer, pH 7.2, and re-suspended in the same buffer. Cell-free extracts were prepared by sonication (20 kHz) six times for 15 s with 30-s intervals and centrifugation (9000*g*, 30 min, 4 °C). The clear supernatant was used as crude extract for enzyme assay (Wojcieszyńska et al. 2011).

Catechol 1,2- and 2,3-dioksygenase activities were estimated by the spectrophotometric method of Hegeman (1966). Catechol 1,2-dioxygenase activity was measured by the formation of *cis,cis*-muconic acid (*ε* = 16,800 dm^3^ · mol^−1^ · cm^−1^), while catechol 2,3-dioxygenase activity was detected by the formation of 2-hydroksymuconic acid (*ε* = 36,000 dm^3^ · mol^−1^ · cm^−1^). The activity of protocatechuate 3,4-dioxygenase was measured by the method described by Hou et al. (1976) and expressed as the amount of oxygen consumption during the oxidation of protocatechuic acid. The specific activities of enzymes were expressed as the number of enzyme units per milligram of protein. The protein concentration in the crude extract was determined by the Bradford method (Bradford 1976) using lysozyme as a standard.

### MIDI-FAME Analysis

The whole-cell-derived fatty acids were extracted in the late exponential phase of growth from bacteria cultured with a single carbon source and on days 1, 4, and 7 from cultures with two carbon sources. Bacteria were harvested by centrifugation (4612*g*, 20 min, 4 °C). The cell pellets were washed with 0.9 % NaCl to remove any residue of the culture medium. Fatty acids were extracted according to the procedure by Sasser (1990) and identified using the Microbial Identification System (MIS; Microbial ID Inc., Newark). FAMEs were separated with a gas chromatograph (Hewlett-Packard 6890) equipped with an HP-Ultra 2 capillary column (25 and 0.22 mm ID) and hydrogen as a carrier gas. FAMEs were detected by a flame ionization detector (FID) and identified using the MIDI Microbial Identification System software (Sherlock TSBA 6.1 method and TSBA6 library; MIDI Inc., Newark, DE, USA).

### Data Analysis

The degradation rate constant (*k*) was determined using the algorithm *C*_*t*_/*C*_0_ = *e*^−*kt*^, where *C*_0_ was the substrate concentration in the culture at time 0 and *C*_*t*_ was the substrate concentration in the culture at time *t*. The average degradation rates (*V*) of aromatic substrates were calculated by dividing the net amount of the degraded compound by the period of time between *t* and 0.

Results were also evaluated by analysis of variance, and statistical analyses were performed on three replicates of data obtained from each treatment. The statistical significance (*p* < 0.05) of differences was treated statistically by two-way ANOVA, considering the effect of substrate and incubation time, and assessed by post hoc comparison of means using the lowest significant difference (LSD) test. The FAME profiles were also subjected to principal component analysis (PCA). This was performed based on the average values of three replicates. All analyses were performed using the Statistica 10.0 PL software package.

## Results

### Degradation of Aromatic Compounds by *Pseudomonas* sp. CF600

The preliminary study showed that *Pseudomonas* sp. CF600 was not able to degrade monochlorophenols at a concentration of 130 mg l^−1^ as a single carbon source in the batch cultures. The depletion of 2-CP, 3-CP, and 4-CP did not exceed 3 % of their initial concentration after 7 days of incubation (data not shown). In connection to this, in the next step, SB, 4-HB, and P were tested as potential growth substrates in the co-metabolic degradation of 2-CP, 3-CP, and 4-CP. As inductors of dioxygenases in bacterial cells, SB, 4-HB, and P were applied. At first, bacteria degraded three successive, identical doses of each aromatic compound as a single carbon source. They metabolized each dose of SB, 4-HB, and P for no more than 9 h. The most rapid degradation was observed in the case of 4-HB (Table 1). As the data obtained from this study indicate, there were significant differences (*p* < 0.05) in the transformation potential of successive dosages of the tested compounds. The highest value of the average rate of disappearance was recorded for the second dosage of 4-HB (130.09 mg h^−1^) and the lowest for the third dosage of P. However, there were no significant differences between *V* values for three dosages of phenol degradation (Table 1). The values of rate constant, following the first-order rate kinetics, were between 0.26 h^−1^ (the third dosage of P) to 0.78 h^−1^ (the first dosage of 4-HB; Table 1).

**Table 1:** Degradation rate constant (*k*) and rate of disappearance (*V*) for SB, 4-HB, and P in batch cultures inoculated with *Pseudomonas* sp. CF600

Substrate	Dosage number	*k*, h^−1^	*V*, mg h^−1^
SB	1	0.36 ± 0.03	68.03 ± 0.57a
	2	0.42 ± 0.10	74.14 ± 5.04b
	3	0.37 ± 0.01	81.69 ± 0.09c
4-HB	1	0.78 ± 0.04a	73.29 ± 2.29a
	2	0.47 ± 0.01b	130.09 ± 14.13b
	3	0.42 ± 0.04b	89.25 ± 4.47a
P	1	0.48 ± 0.00a	50.46 ± 5.61
	2	0.32 ± 0.03b	53.00 ± 6.33
	3	0.26 ± 0.02c	46.79 ± 1.84

The data presented are means of three replicates. The plus/minus values represent standard deviation. The means within each column with different letters are significantly different (*p* < 0.05, LSD test), considering the effect of number of substrate dosages

**Fig. 1:**
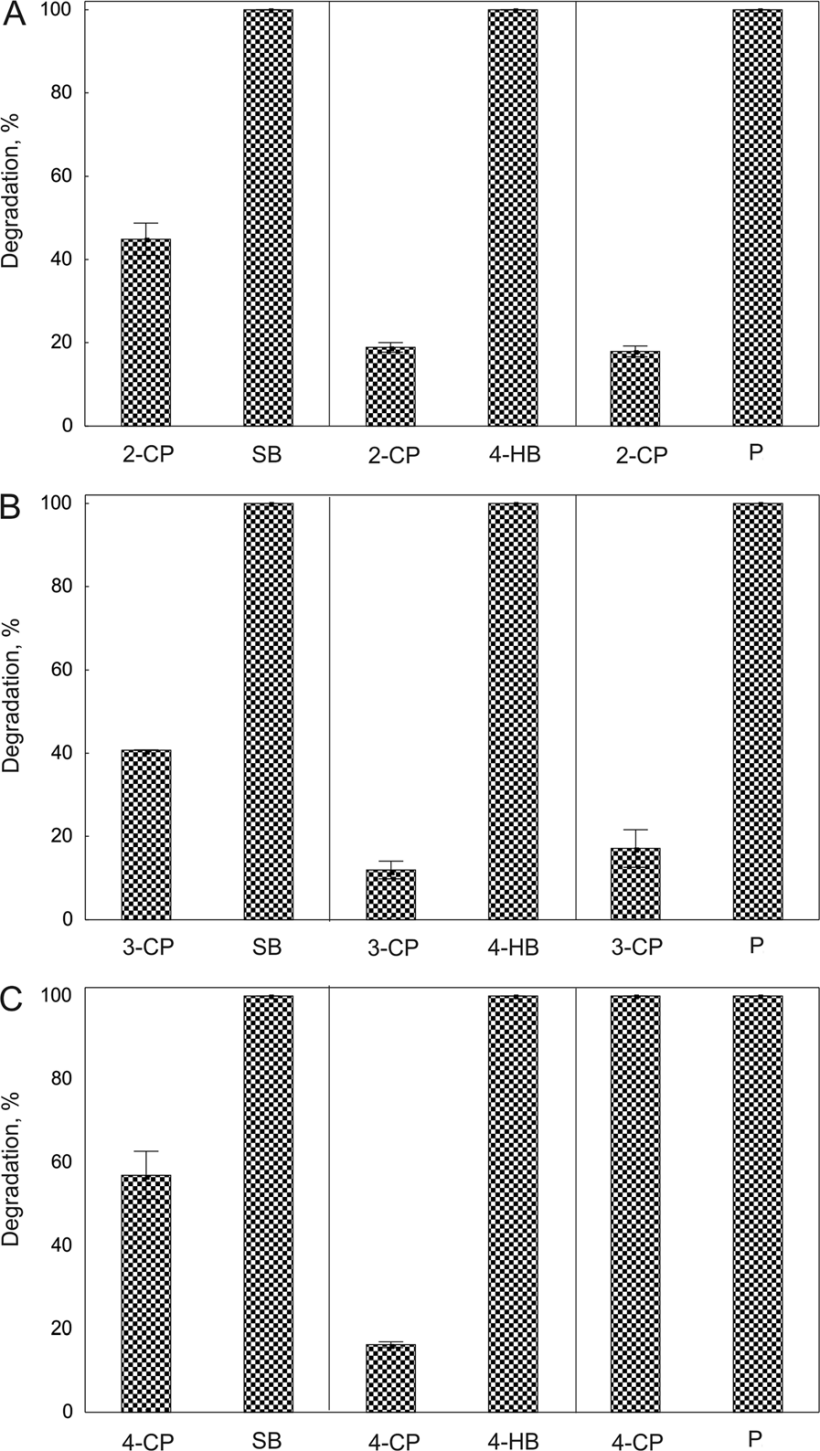
Depletion of 2-CP (**a**), 3-CP (**b**), and 4-CP (**c**) in bacterial culture in the presence of SB, 4-HB, and P during 24 h of incubation. The data presented are means of three replicates

To assess if the presence of an additional carbon source accelerates the degradation of monochlorophenols by *Pseudomonas* sp. CF600 in subsequent studies, bacteria were grown in a medium containing two carbon sources—monochlorophenol (2-CP, 3-CP, or 4-CP) and one of the growth substrates (SB, 4-HB, or P). The highest 2-CP and 3-CP removal was observed in the cultures containing SB as a growth substrate (Fig. 1a, b). During 24 h of incubation, bacteria utilized 45 and 40 % of the initial dose of 2-CP and 3-CP, respectively. At the same time, the depletion of 4-CP in the presence of SB was greater than 2-CP and 3-CP (60 % of 4-CP added), although in the presence of P, bacteria completely degraded 4-CP (Fig. 1c). In turn, in the medium with P, the losses of 2-CP and 3-CP were 18 and 17 %, respectively (Fig. 1a, b). On the basis of the obtained results, for subsequent steps of the study, SB was selected as the additional carbon source for the co-metabolic degradation of all monochlorophenols and P was chosen for the degradation of 4-CP.

**Fig. 2:**
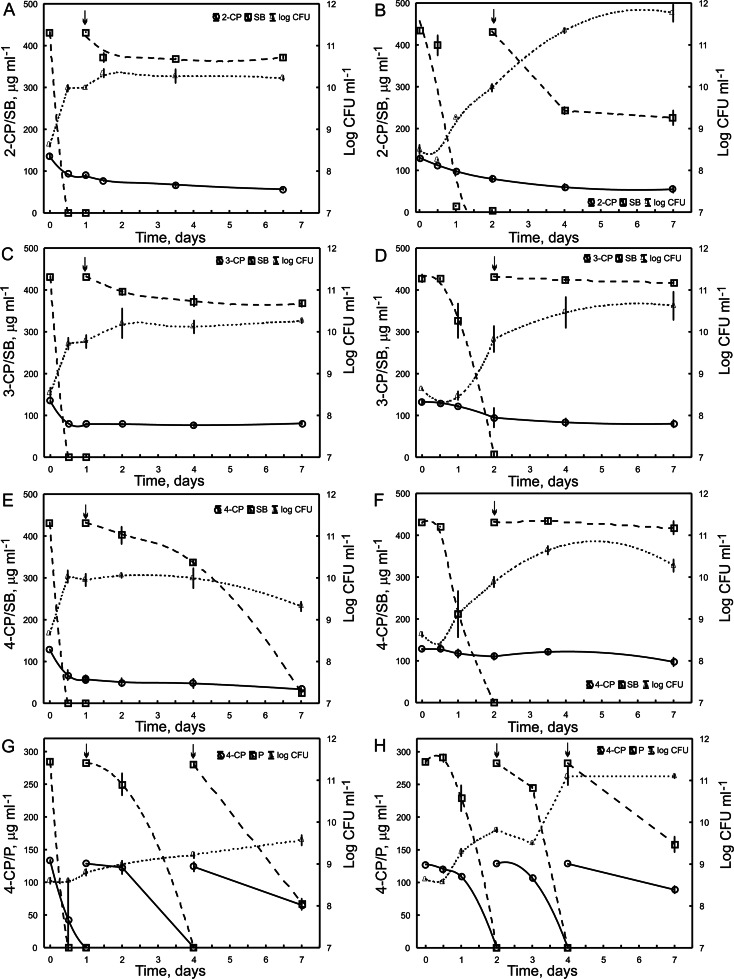
Co-metabolic transformation of 2-CP (**a**, **b**), 3-CP (**c**, **d**), and 4-CP (**e**–**h**) in the presence of SB (**a**–**f**) or P (**g**, **h**) by *Pseudomonas* sp. CF600 and survival of bacteria. **a**, **c**, **e** Transformation by bacteria exposed to SB and **g** transformation by bacteria exposed to P. Each *symbol* represents the mean of three replicates

To estimate the influence of long-term exposure of *Pseudomonas* sp. CF600 to SB and P on its ability to co-metabolically degrade monochlorophenols, cells were cultured in a medium containing a mixture of each monochlorophenol and SB, as well as 4-CP and P for 7 days. In the case of each substrate depletion, its identical dosage was added to the medium. Cells that degraded three successive doses of SB or P were treated as exposed cells. It was found that the highest removal of aromatic compounds was detected in cultures with exposed cells during the first day of incubation (Fig. 2a, c, e, g). Among all monochlorophenols, only 4-CP in the presence of P was completely metabolized in that time. The second dosage of 4-CP and P bacteria were completely removed during the next 3 days (Fig. 2g). Within the next 3 days, 50 % of the third dosage of 4-CP and 77 % of P added to the medium were removed. In comparison, cells exposed to SB were not able to completely degrade monochlorophenols during 1 week of incubation. At that time, bacteria transformed 62, 40, and 75 % of 2-CP, 3-CP, and 4-CP, respectively (Fig. 2a, c, e). Data obtained from a parallel study on the co-metabolic degradation of monochlorophenols by *Pseudomonas* sp. CF600 unexposed to SB nor P indicated the lower degradative potential of such cells toward 2-CP and 4-CP in comparison with exposed cells. Unexposed bacteria in the presence of SB transformed 50 % of the initial dosage of 2-CP (Fig. 2b) and 23 % of 4-CP (Fig. 2f) within 7 days of incubation. In turn, in the presence of P, they degraded two doses of 4-CP within 4 days and during the following 3 days, utilized 30 % of the third dose (Fig. 2h). Both cells unexposed and exposed to SB removed 40 % of the initial dose of 3-CP during 7 days (Fig. 2c, d). Interestingly, the numbers of bacteria in cultures unexposed to SB or P were higher in comparison with their numbers in cultures with exposed bacteria. The highest bacterial counts were estimated in the medium inoculated with unexposed cells in the presence of 2-CP and SB. The number of cells increased in this culture by 40 % compared to the initial counts during 1 week of incubation (Fig. 2b). At the same time, the number of bacteria in the medium inoculated with cells exposed to SB and amended with 2-CP increased by 20 % in comparison with initial counts (Fig. 2a).

### Activity of Dioxygenases

Parallel to the degradation study, the activities of catechol 1,2-dioxygenase, catechol 2,3-dioxygenase, and protocatechuate 3,4-dioxygenase in *Pseudomonas* sp. CF600 were determined. It was evidenced that the specific activities of all enzymes tested increased during the degradation of successive doses of SB, 4-HB, and P. The highest activity of protocatechuate 3,4-dioxygenase (1488.52 mU mg^−1^ protein) bacteria was exhibited during degradation of the third dosage of 4-HB. Catechol 1,2-dioxygenase was induced in cells by SB, and it showed the highest activity (120.49 mU mg^−1^) during the degradation of the third dose of this substrate. In turn, in the presence of P and SB, *Pseudomonas* sp. CF600 exhibited catechol 2,3-dioxygenase. Its activity in bacteria degrading the third dosage of P was 100-fold higher in comparison with the activity of this enzyme in cells growing in the presence of SB (Table 2).

**Table 2:** Activities of dioxygenases of *Pseudomonas* sp. CF600 during degradation of aromatic substrates

	Specific activity, mU mg^−1^ protein								
	Catechol 1,2-dioxygenase			Catechol 2,3-dioxygenase			Protocatechuate 3,4-dioxygenase		
Inductor	SB	4-HB	P	SB	4-HB	P	SB	4-HB	P
Dosage 1	20.80 ± 2.44a	0.00 ± 0.00	0.00 ± 0.00	20.95 ± 1.84a	0.00 ± 0.00	215.55 ± 2.38a	0.00 ± 0.00	495.53 ± 32.11a	0.00 ± 0.00
Dosage 2	67.50 ± 2.87b	0.00 ± 0.00	0.00 ± 0.00	30.35 ± 3.37b	0.00 ± 0.00	280.13 ± 16.57b	0.00 ± 0.00	505.78 ± 59.49a	0.00 ± 0.00
Dosage 3	120.49 ± 6.36c	0.00 ± 0.00	0.00 ± 0.00	4.48 ± 0.63c	0.00 ± 0.00	474.66 ± 16.07c	0.00 ± 0.00	1488.52 ± 66.96b	0.00 ± 0.00

The data presented are means of three replicates. The plus/minus values represent standard deviation. The different letters indicate significant differences (*p* < 0.05, LSD test), considering the effects of aromatic compound dosage

The specific activities of the tested dioxygenases were also determined under co-metabolic conditions. As shown in Table 3, monochlorophenols did not influence catechol 1,2-dioxygenase activity. It turn, the activity of protocatechuate 3,4-dioxygenase decreased by 40 % in cells growing in the presence of monochlorophenols and 4-HB in comparison with its activity when bacteria were cultured with 4-HB only. However, monochlorophenols affected the catechol 2,3-dioxygenase activity. In the presence of 2-CP, 3-CP, and 4-CP, the decline of this enzyme activity was about 80, 65, and 50 %, respectively, as compared to activity detected in cells in the presence of P (Table 3).

**Table 3:** Relative activity of dioxygenases of *Pseudomonas* sp. CF600 during transformation of 2-CP, 3-CP, and 4-CP in the presence of SB, 4-HB, and P

Substrate(s)	Relative activity, %
Catechol 1,2-dioxygenase	
SB	100.00 ± 8.44ab
SB + 2-CP	111.31 ± 6.89a
SB + 3-CP	87.56 ± 5.87b
SB + 4-CP	96.06 ± 18.19ab
Protocatechuate 3,4-dioxygenase	
4-HB	100.00 ± 10.94a
4-HB + 2-CP	61.06 ± 1.78b
4-HB + 3-CP	65.11 ± 4.95b
4-HB + 4-CP	62.56 ± 0.92b
Catechol 2,3-dioxygenase	
P	100.00 ± 4.91a
P + 2-CP	20.41 ± 1.61b
P + 3-CP	34.19 ± 2.49bc
P + 4-CP	47.89 ± 4.63c

The data presented are means of three replicates. The plus/minus values represent standard deviation. The different letters indicate significant differences (*p* < 0.05, LSD test), considering the effects of monochlorophenols on enzymes activity

### FAME Analysis

**Fig. 3:**
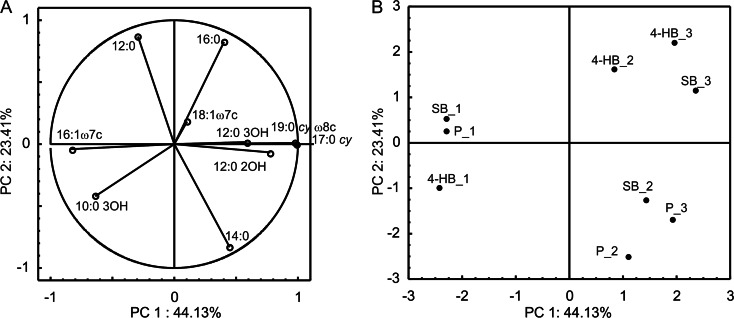
PCA of analyzed FAMEs during degradation of three successive doses of SB (SB_1, SB_2, and SB_3), 4-HB (4-HB_1, 4-HB_2, and 4-HB_3), and P (P_1, P_2, and P_3) by *Pseudomonas* sp. CF600. **a** Correlation of the fatty acids with PC1 and PC2 and **b** projection of FAMEs on the plane defined by PC1 and PC2

To illustrate FAME variability in *Pseudomonas* sp. CF600 cells during the degradation of successive doses of SB, 4-HB, or P, whole-cell-derived fatty acids were directly extracted from bacteria. The PCA of the FAME patterns involved the most common fatty acids (Fig. 3a). FAMEs isolated from bacteria during the degradation of the first dosage of SB, 4-HB, and P were clearly distinguished along the first axis from FAMEs isolated during the degradation of the second and the third dosages of these substrates (Fig. 3b). Simultaneously, FAME profiles obtained during the degradation of the second and the third dosages of 4-HB and the third dosage of SB were distinguished along the second axis from profiles obtained during the degradation of the second and the third dosages of P and the second dosage of SB (Fig. 3b). It should be noted that during the degradation of successive doses of the tested substrates, the percentage of 16:1 ω7*c* in FAME profiles decreased, although the content of cyclopropane (17:0 *cy* and 19:0 *cy* ω8*c*) and hydroxylated (12:0 2OH and 12:0 3OH) fatty acids increased. Furthermore, during the degradation of successive doses of 4-HB, the percentage of 16:0 fatty acid increased, while during the degradation of P, the content of 14:0 increased (Fig. 3a, b).

**Fig. 4:**
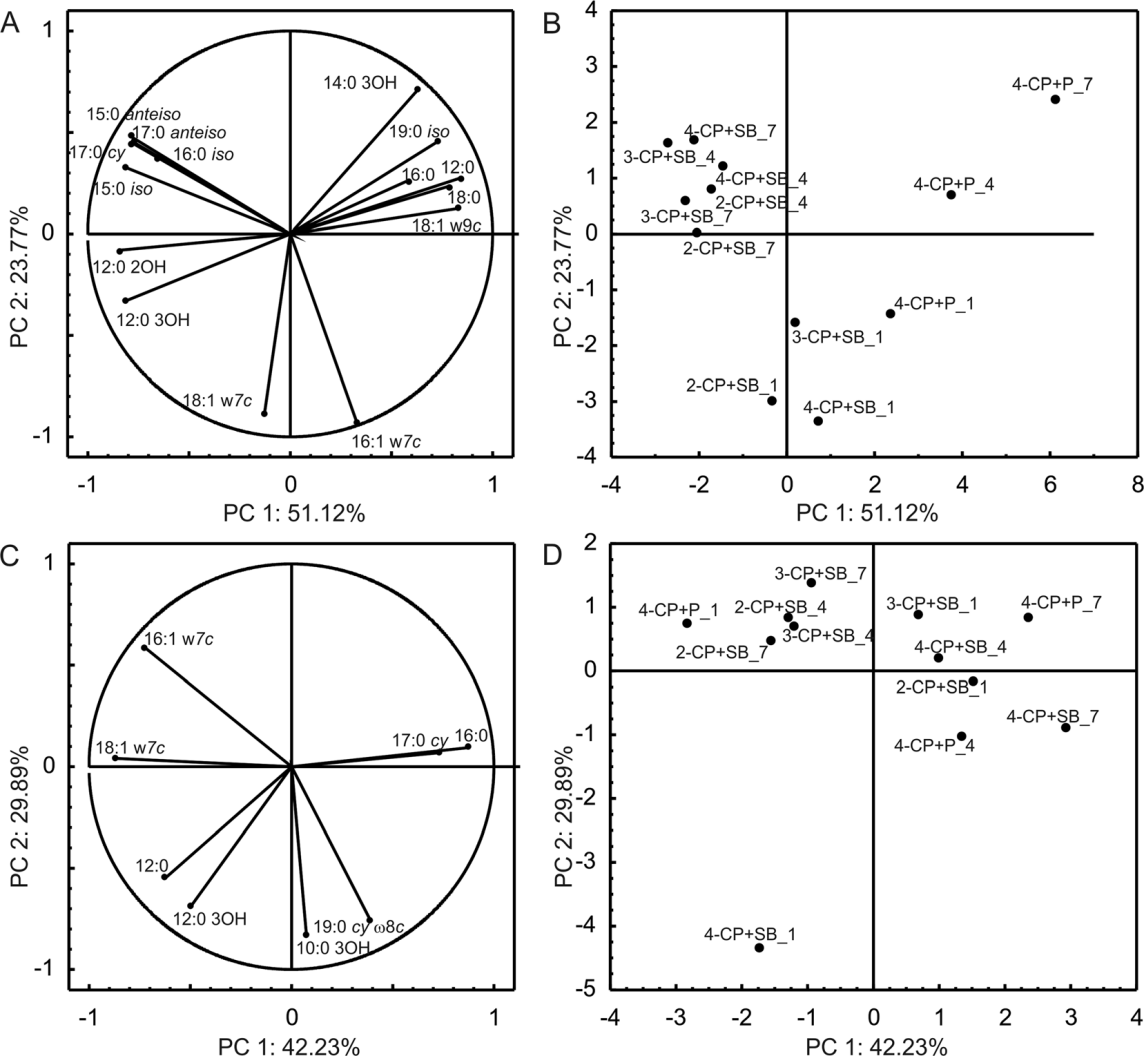
PCA of analyzed FAMEs during co-metabolic transformation of monochlorophenols in the presence of SB or P by *Pseudomonas* sp. CF600 unexposed (**c**, **d**) and exposed (**a**, **b**) to SB or P. **a**, **c** Correlation of fatty acids with PC1 and PC2. **b**, **d** Projection of FAMEs on the plane defined by PC1 and PC2. *2-CP + SB_X* FAMEs isolated from bacteria degrading 2-CP and SB, *3-CP + SB_X* FAMEs isolated from bacteria degrading 3-CP and SB, *4-CP + SB_X* FAMEs isolated from bacteria degrading 4-CP and SB, *4-CP + P_X* FAMEs isolated from bacteria degrading 4-CP and P, and *X* sampling day

FAMEs were also isolated from *Pseudomonas* sp. CF600 cells unexposed and exposed to SB and P during the co-metabolic degradation of monochlorophenols. The PCA of FAMEs obtained from cells exposed to SB or P involved 15 fatty acids (Fig. 4) and from unexposed cells involved 8 fatty acids (Fig. 4c). The PCA analysis distinguished FAMEs isolated from cells exposed to P during the degradation of the mixture of 4-CP and P on all sampling days along the first axis from FAMEs obtained from bacteria cultured with monochlorophenols and SB on days 4 and 7 (Fig. 4b). Simultaneously, all FAMEs extracted from bacteria on day 1 were distinct in comparison with the other fatty acids along the second axis (Fig. 4b). On the basis of the obtained results, it should be evidenced that *Pseudomonas* sp. CF600 under exposure to aromatic compounds showed an increase in the degree of saturation of fatty acids. The percentage of saturated FAMEs increased during the co-metabolic degradation of monochlorophenols, although the content of unsaturated fatty acids (16:1 ω7*c* and 18:1 ω7*c*) decreased. Furthermore, it was also demonstrated that in the presence of SB as a growth substrate, the percentage of branched (15:0 *iso*, 15:0 *anteiso*, 16:0 *iso*, and 17:0 *anteiso*) and cyclopropane (17:0 *cy*) fatty acids increased, while in the presence of P, the content of straight-chain fatty acids mainly increased (12:0, 16:0, and 18:0; Fig. 4a, b). In FAME profiles of cells unexposed to SB or P but degrading monochlorophenols co-metabolically, the most distinct were FAME profiles of cells degrading 4-CP with SB or P (Fig. 4d). These profiles obtained from bacteria on day 1 were characterized by a higher percentage of unsaturated fatty acids in comparison with profiles on days 4 and 7 of the experiment, when the increase of 17:0 *cy* and 16:0 fatty acid content was recorded (Fig. 4c, d). The other FAME profiles were gathered together (Fig. 4d).

## Discussion

Monochlorophenols belong to aromatic compounds with a chlorine substituent. The presence of this electron-withdrawing group on the aromatic ring makes such compounds more resistant to microbial degradation and more toxic for living organisms than un-substituted analogues (Escuder-Gilabert et al. 2001; Chrzanowski et al. 2011). One of the possibilities of removing these compounds from contaminated environments is co-metabolic degradation by microorganisms (Kim and Hao 1999; Lee and Lee 2007). In this study, their degradation by *Pseudomonas* sp. CF600 in the presence of selected compounds was investigated. As co-substrates, phenol, sodium benzoate, and 4-hydroxybenzoic acid were used. P was chosen as a structural analogue of monochlorophenols and frequent contaminant of soil and industrial wastes. Sodium benzoate (BS) is less toxic than phenol and has low potential to accumulate in living organisms (WHO 2000). In turn, 4-HB is mainly known as an allelochemical secondary plants metabolite and, similarly to sodium benzoate, is not regarded as a contaminant (Wu et al. 2009). The co-metabolic degradation of monochlorophenols by bacteria was studied in the presence of glucose, sodium glutamate, and phenol as growth substrates (Wang and Loh 2000; Tarighian et al. 2003). However, there is a lack of reports about the use of sodium benzoate and 4-hydroxybenzoic acid as co-substrates. Greń et al. (2010) indicated that benzoic acid, 4-hydroxybenzoic acid, and 3,4-dihydroxybenzoic acid stimulated the biotransformation of mononitrophenols by *Stenotrophomonas maltophilia* KB2. These plant origin aromatic compounds induced catechol and protocatechuate dioxygenases involved in a broad range of aromatic compounds’ degradation pathways. The un-specificity of these enzymes permit to co-metabolize compounds, which are barely biodegradable as sole carbon sources (Bugg and Ramaswamy 2008; Guzik et al. 2009, 2013). The concentrations of monochlorophenols added to the culture medium had mean values in comparison with concentrations used by other authors (Kim and Hao 1999; Wang and Loh 2000; Lee and Lee 2007).

The degradation study of the following doses of SB, 4-HB, and P by *Pseudomonas* sp. CF600 indicated that bacteria required the longest time to metabolize the first doses of these compounds, which could be connected with the induction of enzymes of adequate catabolic pathways. Among all the tested substrates, the rate of disappearance of 4-HB was the highest and the loss of P was the lowest. These differences may be connected with their toxicity to bacterial cells. Moreover, the higher value of *V* for 4-HB in comparison with SB could be related with the presence of an additional hydroxyl group in the 4-HB structure which facilitates the aromatic ring cleavage (Greń et al. 2010). Similarly, the numbers of bacteria were the lowest during the degradation of the first dosage of SB, 4-HB, and P, as compared to their numbers during the degradation of subsequent doses.

The activities of three various dioxygenases detected during the degradation of SB, 4-HB, and P indicated that *Pseudomonas* sp. CF600 has three different metabolic pathways of aromatic compound degradation. In the presence of SB, bacteria exhibited high activity of catechol 1,2-dioxygenase and the slight activity of catechol 2,3-dioxygenase. The lack of activity of protocatechuate 3,4-dioxygenase during SB degradation may suggest that *Pseudomonas* sp. CF600 degraded SB through the catechol pathway with the simultaneous induction of additional decarboxylase (Peng et al. 2003; Wojcieszyńska et al. 2011). Furthermore, it was found that this strain metabolized P via the *meta*-cleavage pathway, which is consistent with data obtained by Powlowski and Shingler (1994). It should also be noted that this is the first report about the activity of intradiol protocatechuate 3,4-dioxygenase in *Pseudomonas* sp. CF600.

The obtained results indicated that *Pseudomonas* sp. CF600 was not able to remove monochlorophenols completely in the presence of SB or 4-HB. Despite the fact that bacteria degraded 4-HB as a sole carbon source in the shortest time and synthesized protocatechuate 3,4-dioxygenase, 4-HB was not an appropriate growth substrate for the co-metabolic degradation of monochlorophenols. In turn, the removal of 2-CP and 3-CP by *Pseudomonas* sp. CF600 was most effective in the presence of SB, although the degradation of these co-metabolites was incomplete. Among the tested isomers, bacteria were capable of degrading only 4-CP completely in the presence of P. This could be connected with the similar structure of these compounds, as well as the position of the chloride atom on the aromatic ring. Menke and Rehm (1992) reported that the substitution of the second hydroxyl group to the aromatic ring in 4-CP is easier than the substitution of 2-CP and 3-CP.

As described in results, bacteria exposed to SB or P removed 2-CP and 4-CP more effectively than unexposed ones. This may suggest that the exposition of bacteria used in the bioaugmentation of contaminated environments can accelerate the removal of xenobiotics and reduce the time of their biodegradation. The lack of differences in the degradation of 3-CP by unexposed and exposed bacteria probably resulted from the unfavorable configuration of hydroxyl and chloride substituents on the aromatic ring. This configuration affected the introduction of a second hydroxyl group onto the aromatic ring and diminished the enzymatic transformation of 3-CP (Menke and Rehm 1992).

Analyses of enzymes’ activities could suggest that 4-CP was metabolized by *Pseudomonas* sp. CF600 via the *meta*-cleavage pathway, even though catechol 2,3-dioxygenase was the most sensitive to monochlorophenols in comparison with catechol 1,2-dioxygenase and protocatechuate 3,4-dioxygenase. It was also proved that the activity of catechol 1,2-dioxygenase was not inhibited by 2-CP, 3-CP, and 4-CP. The complete inhibition of catechol 1,2-dioxygenase, catechol 2,3-dioxygenase, and protocatechuate 3,4-dioxygenase in the presence of mononitrophenols was reported by Wojcieszyńska et al. (2011). The higher sensitivity of dioxygenases to mononitrophenols in comparison with monochlorophenols was likely to be connected with the various toxicities of these phenol derivatives (Michałowicz and Duda 2007). On the other hand, the presence of monochlorophenols did not inhibit the degradation of SB, 4-HB, and P by *Pseudomonas* sp. CF600 during incubation in the co-metabolic culture. The increase in the number of cells, especially unexposed to SB, 4-HB, or P, did not indicate the significant influence of monochlorophenols on their survival.

Our results demonstrated that *Pseudomonas* sp. CF600 underwent structural changes of cell-derived fatty acids when grown on SB, 4-HB, or P. The highest variability in FAME composition was observed during the degradation of the first and the second dosages of each compound. These changes involved alterations in the degree of saturation of fatty acids, hydroxylation, and cyclopropane ring formation. The increasing degree of membrane saturation is a major adaptive mechanism that enables bacterial cells to survive under aromatic hydrocarbon stress (Segura et al. 2010). It was indicated that changes in the ratio of straight-chain and hydroxylated fatty acids in cellular lipids permitted the regulation of the uptake of amphipathic compounds, reducing their toxic effect. The increase of straight-chain fatty acid content caused the reduction of polar substance uptake. The presence of hydroxy fatty acids caused the opposite effect (Donato et al. 1997; Kaczorek et al. 2013). Another mechanism of bacteria adaptation to SB, 4-HB, and P was the increase of the content of cyclopropane fatty acids. The cyclopropane ring is less reactive than the double bond of their precursors—*cis*-unsaturated fatty acids, and decreased the fluidity of the membrane. An increase of cyclopropane fatty acid content in Gram-negative bacteria was observed in various stress conditions (Chang and Cronan 1999; Mrozik et al. 2004; Muñoz-Rojas et al. 2006).

Interestingly, the earlier exposition of the bacteria tested to SB and P had an influence on the diversity of fatty acids during the co-metabolic degradation of monochlorophenols. The main difference was the appearance of branched fatty acids in bacteria exposed to SB on days 4 and 7 of the experiment. The absence of these fatty acids in FAMEs isolated from bacteria during the degradation of SB as the sole carbon source suggested that branched fatty acids were involved in the adaptation of bacteria to monochlorophenols. Despite the fact that branched fatty acids are regarded as signature lipid biomarkers of Gram-positive bacteria (Piotrowska-Seget and Mrozik 2003), their content in Gram-negative bacteria under stress conditions could also be significant in *Pseudomonas putida* and *Pseudomonas stutzeri* exposed to naphthalene (Mrozik et al. 2005). The main alteration in fatty acid composition observed in *Pseudomonas* sp. CF600 degraded 4-CP, and P was the increase of the content of straight-chain fatty acids. It should be pointed out that in bacteria unexposed to SB and P, distinct adaptive mechanisms were observed only during the degradation of 4-CP and SB, as well as 4-CP and P. In FAMEs isolated from bacteria on days 4 and 7, significant increases of straight-chain (16:0) and cyclopropane (17:0 *cy*) fatty acid content were recorded. During the co-metabolic degradation of 2-CP and 3-CP by unexposed bacteria, well-defined changes in their fatty acid profiles were difficult to find.

## Conclusions

The obtained results indicate that phenol was the most suitable growth substrate in the degradation of monochlorophenols by *Pseudomonas* sp. CF600 in comparison with sodium benzoate and 4-hydroxybenzoic acid. However, among tested enzymes, the activity of catechol 2,3-dioxygenase induced by phenol was the most inhibited by monochlorophenols. It was proved that chlorophenols in the co-metabolic cultures caused additional alterations in whole-cell fatty acid composition of bacteria as compared to changes induced in the cells by the growth substrate only.

It should be emphasized that the co-metabolic degradation of monochlorophenols by the *Pseudomonas* sp. CF600 strain has not yet been studied. This study confirmed that it was able to degrade 4-CP and P completely, which makes it a good candidate for the bioaugmentation of contaminated areas, where both compounds are frequently present as a mixture. Additionally, its capability to degrade 2-CP and 3-CP partially in the presence of additional growth substrates makes it possible to accelerate the biodegradation processes in specific cases. Moreover, the analysis of fatty acid composition expanded our knowledge on the adaptive mechanisms of cells exposed to highly toxic and persistent chlorophenols.
